# Lactic acid bacterial surface display of scytovirin inhibitors for anti-ebolavirus infection

**DOI:** 10.3389/fmicb.2023.1269869

**Published:** 2023-11-23

**Authors:** Joshua Wiggins, Ngan Nguyen, Wenzhong Wei, Leah Liu Wang, Haley Hollingsead Olson, Shi-Hua Xiang

**Affiliations:** ^1^Nebraska Center for Virology, Lincoln, NE, United States; ^2^School of Biological Sciences, University of Nebraska-Lincoln, Lincoln, NE, United States; ^3^School of Veterinary Medicine and Biomedical Sciences, University of Nebraska-Lincoln, Lincoln, NE, United States

**Keywords:** scytovirin, lectins, lactic acid bacteria, Ebola virus, bacterial engineering, surface display

## Abstract

Scytovirin (SVN) is a lectin from cyanobacteria which has a strong inhibitory activity against Ebola virus infection. We engineered scytovirin as the inhibitor for surface display of lactic acid bacteria to block Ebola virus infection. Two different bacterial strains (*Lactobacillus casei* and *Lactococcus lactis*) were successfully engineered for scytovirin expression on the bacterial surface. These bacteria were found to be effective at neutralizing pseudotyped Ebolavirus in a cell-based assay. This approach can be utilized for prophylactic prevention, as well as for treatment. Since lactic acid bacteria can colonize the human body, a long-term efficacy could be achieved. Furthermore, this approach is also simple and cost-effective and can be easily applied in the regions of Ebola outbreaks in the developing countries.

## 1 Introduction

Ebola virus (EBOV) is an enveloped negative-stranded RNA virus which can cause severe Ebola viral disease (EVD) in humans and nonhuman primates (Jacob et al., 2020). EBOV belongs to the family *Filoviridae* along with Marburg virus that also causes a similar disease. Ebolavirus in Zaire was first identified in 1976 in Africa (Bowen et al., 1980), now five Ebola species have been recognized (Kuhn et al., 2019): Zaire ebolavirus (ZEBOV or EBOV), Sudan ebolavirus (SUDV), Tai Forest ebolavirus (TAFV), Reston ebolavirus (RESTV) and Bundibugyo ebolavirus (BDBV). Ebolavirus has produced more than 20 outbreaks in humans with high mortality rates from 25 to 90% (Feldmann and Geisbert, 2011;WHO, n.d.). The recent large Ebola outbreak in 2014 in West Africa infected more than 28,000 people and more than 11,000 died (Cenciarelli et al., 2015). It is evident that this emerging and reemerging viral pathogen represents a great threat to human health. However, we do not have any medicines to treat this lethal viral disease until December 2019, when a vaccine (rVSVΔG-ZEBOV-GP) was approved by FDA for limited use against Zaire Ebolavirus, and in 2020, two antibody drugs (mAb114 and REGN-EB3) were approved for treating this viral infection. It is apparent that more medicines are required for fighting against this deadly, infectious viral disease.

Scytovirin (SVN) is a small protein of 95 amino acids which was first identified from cyanobacteria of *Scytonema* var*ium* (Bokesch et al., 2003). Scytovirin is a type of lectin which are known carbohydrate-binding proteins with non-immunologic nature (Fernandez Romero et al., 2021). Because lectins recognize the carbohydrates on the glycoproteins of viral particles, they usually exhibit antiviral activities (Akkouh et al., 2015; Naik and Kumar, 2022). Lectins from algae, plants and cyanobacteria show strong inhibitory activity against HIV infection (Li et al., 2008; Akkouh et al., 2015). Due to their inhibitory effect and immunosuppressive properties, lectins such as cyanovirin-N (CV-N) (Colleluori et al., 2005; Li et al., 2011; O'Keefe et al., 2015; Vamvaka et al., 2016) and griffithsin (GRFT) (Emau et al., 2007; Girard et al., 2018; Alexandre et al., 2020), have been used as microbicides for treating viral diseases (Abdool Karim and Baxter, 2012; Huskens and Schols, 2012; Koharudin and Gronenborn, 2014) and have been investigated for pre-exposure prophylaxis (PrEP) against HIV-1 infection. Like the well-studied lectins CV-N and GRFT in HIV research, SVN has also shown highly specific activity to the high mannose moieties that exist on the surface of glycoproteins of HIV, Ebola, and Marburg viruses (Barrientos and Gronenborn, 2005, Mori et al., 2005, Alexandre et al., 2010). More studies have demonstrated that scytovirin has much stronger activity against Ebola than HIV *in vitro* and *in vivo* (Garrison et al., 2014).

Because scytovirin demonstrated potent antiviral activity against Ebola infection, we used it to develop a novel live microbicide for Ebola infection control. This approach displayed scytovirin on the surface of Lactic acid bacteria (LAB) for delivery against Ebola virus infection. These bacteria can be delivered into mucosal surfaces of the body (e.g., mouth, nose, and GI tract) that are the ports of viral entry where they can colonize and replicate. Previous studies using LAB expressing the antiviral lectin CV-N demonstrated that the bacteria can pass through the GI tract unharmed and stably produce lectins to inhibit HIV (Lagenaur et al., 2011, Li et al., 2011, Brichacek et al., 2013). Therefore, this approach for application of viral inhibition should be safe, long-lasting and effective. Furthermore, this approach is cost-effective and easy-to-use, so it is especially valuable for use in those outbreak regions in Africa. Here, we reported the *in vitro* data we have achieved successfully in this research direction.

## 2 Methods and materials

### 2.1 Strains, cell lines, and plasmids

*Escherichia coli DH5α* and *E. coli BL21 DE3* were used for cloning and initial protein verification, respectively. Both were cultured in LB media (FisherSci, BP1427-500) overnight in a shaker at 250 rpm and 37°C. The antibiotics used for plasmid selection were 50 μg/mL kanamycin for pET28a and 250 μg/mL erythromycin for pLSVN3 and pLSVN7 (Table 1). *L. casei* and *L. lactis* were cultured statically in MRS media (FisherSci, CM0359) at 37°C and 5% CO2 for 2–3 days until reaching log phase. For engineered bacterial culturing, 5 μg/mL erythromycin was added to the MRS media for applying selection pressure. HEK 293 T and TZM-bl mammalian cell lines were grown in Dulbecco Minimum Essential Media (DMEM) (Gibco, 11,965,092) supplemented with 10% FBS (Gibco, 10,082), 1 mM L-glutamine (Gibco, 25,030), and 100 μg/mL penicillin/streptomycin (Gibco, 15,140). Cells were cultured in a humidified cell culture incubator at 37°C with 5% CO2 using T-75 flasks.

**Table 1:** List of strains, cell lines, plasmids, and primers used in this study.

Materials	Relevant characteristics	Source or reference
Strains		
*Lactobacillus casei*	host strain for SVN engineering	ATCC
*Lactococcus lactis*	host strain for SVN engineering	ATCC
*E. coli BL21 DE3*	For SVN expression	Previous study
*E. coli DH5α*	For plasmid amplification	Previous study
Cell lines		
HEK 293 T	human embryonic kidney 293 cells for making pseudotyped viruses	Previous study
TZM-bl	HIV permissive cells under Tat-responsive long terminal repeat (LTR) promoter driving the expression of firefly luciferase and beta-galactosidase used as report cells.	NIH HIV Research reagents program
Plasmids		
pSG3^ΔEnv^	An envelope gene defective HIV clone (cat no. ARP-11051, GenBank: L02317) used as the backbone for pseudotyping viruses.	NIH HIV Research reagents program
pWZ486	pTRKH-Pldh-SP1-GFP-CD4-ANC, Erm^r^	Previous study (Wei et al., 2019)
		
pET28a-SVN	for SVN-E-tag expression in *E. coli*	This study
pLSVN3	for *Lactobacillus casei* engineering	This study
pLSVN7	for *Lactococcus lactis* engineering	This study
Primers		
037	GCGCCTGCAGCTATTCTTCACGTTGTTTCCGTTTC	ANC, reverse
048	GCGCGAATTCGCAGTCGACAAGCTTTTTAGTC	*Pldh*, forward
057	AATTCTCGAGTCCTGAGCCTTTGTATAGTTCATCCATG	GFP, reverse
141	GCGACTCGAGGATAAGAAGACTTCGCTGC	ANC, forward
154	GCAGCAACCATAGAAAGCGGAGGTAGTAAAGGAGAAG	*Pldh*-SP-GFP, forward
155	CTTCTCCTTTACTACCTCCGCTTTCTATGGTTGCTGC	*Pldh*-SP-GFP, reverse
198	GCGCGAGCTCATGGGAGCACCACTACC	SVN Forward primer to test integration
199	GCGCCTGCAGCTACTCGAGTGCGG	SVN Reverse primer to test integration

Erm^r^, erythromycin resistance gene; SP, signal peptide; GFP, green fluorescent protein, ANC, anchor (prtP) (Wei et al., 2019). SVN, scytovirin; Restriction enzymatic sites are underlined.

### 2.2 Scytovirin gene expression in *E. coli*.

The scytovirin (SVN) gene sequence (GenBank: P86041.1) (Bokesch et al., 2003) with an added E-tag sequence (total 348 bp) was synthesized with *SacI*/*XhoI* restriction sites by GenScript and inserted into the vector pET28a (Novagen, Inc.) to test protein expression in *E. coli BL21 (DE3)* bacterial cells. SVN was overexpressed in *E. coli* previously by Xiong et al. (2006). SVN expression was induced by IPTG (1 mM) to increase protein production which was verified by Western blotting using an anti-E-tag (ab3397, Abcam), anti-His-tag (HRP-66005, Proteintech) or anti-SVN (A64238-050, Epigentek) polyclonal antibodies.

### 2.3 Scytovirin constructs for surface display on LAB

To express the SVN protein on the surface of LAB, constructs were created which included the *Lactate dehydrogenase* promoter (*Pldh*), signal peptide (SP), cell membrane anchor protein (ANC), E-tag marker (E), and the protein marker (GFP). The constructs were built up based on our previous plasmid pWZ486 (Wei et al., 2019) derived from pTRKH3-ldhGFP (Addgene). The SVN-E-tag-GFP fusion sequence replaced the CD4 gene sequence in pWZ486 using the *SacI/XhoI* restriction sites. The constructs pLSVN3 and pLSVN7 were verified by DNA sequencing and PCR using the specific primers (Table 1) marked in the construct maps.

### 2.4 Electroporation

Plasmids were transformed into the Lactic acid bacteria (LAB) by electroporation as previously described (Wei et al., 2019; Welker et al., 2019). Briefly, overnight cultures of LAB cells were diluted (1:50) into fresh MRS media with 1% glycine and incubated at 37°C without shaking for 2 h. Cells were harvested and treated with 50 mM EDTA (pH 8.0) for 15 min, followed by two washes with ice-cold electroporation buffer (0.5 M sucrose) and resuspended in electroporation buffer (1/100 volume of the initial culture). 50 μL of cells were mixed with plasmid DNA and incubated on ice for 15 min. The mixture was added to an ice-cold 0.2 cm GenePulser (Biorad) cuvette and pulse was immediately applied at the conditions of 10 KV/cm, 200 Ω, and 25 μF. Cells were suspended in 1 mL MRS broth with 2 mM CaCl2 and 20 mM MgCl2 and then incubated at 37°C for 4 h. Cells were pooled on MRS plates with 5 μg/mL erythromycin and cultured as described above. To verify transformation, single colonies were picked and added to 25 μL PCR master mix (Promega, M791B) containing 1 μM forward and reverse primers (Table 1). PCR amplification occurred in a SimpliAmp thermocycler (Applied BioSystems, A24811) under the following conditions: Stage 1; 95°C for 5 min, Stage 2 (35 cycles); 95°C for 30 s, 55°C for 30 s, 72°C for 1 min, Stage 3; 72°C for 7 min. PCR products were visualized on 1% agarose gel electrophoresis.

### 2.5 Flow cytometry

For the pLSVN3 construct transformed into *L. casei*, the fusion protein was detected based on the GFP fluorescence. Bacteria were washed three times with PBS and analyzed on a BD FACSAria using a 488 nm laser. For the pLSVN7 construct transformed into *L. lactis*, the bacteria were first stained for 1 h with two primary antibodies: mouse monoclonal anti E-tag (Novus NBP2-67081) and rabbit polyclonal anti SVN (A64238-050, Epigentek). Goat anti mouse conjugated with AlexaFluor488 (A-11011, ThermoFisher) and goat anti rabbit conjugated with AlexaFluor594 (A-11012, ThermoFisher) were used as secondary antibodies, respectively. Bacteria were analyzed on a Beckman Coulter CytoFLEX FX at 488 nm and 561 nm. Unstained bacteria and stained wild-type bacteria were used in all experiments for an appropriate gating strategy.

### 2.6 Confocal microscopy

Confocal microscopy was performed using the same fluorescent fusion protein or antibody combinations as described for flow cytometry (above). Following the antibody staining, bacteria were pelleted, resuspended in 10 μL PBS, and transferred to a microscope slide with cover slip. Images were captured using a Nikon A1R-Ti2 (Nikon Instruments, NY, USA) inverted confocal system.

### 2.7 Pseudotyping viruses

The Ebola pseudotyped viruses were made from a HIV-1 backbone plasmid, pSG3^ΔEnv^ (NIH HIV Reagent Program). The Ebola Envelope gene (GP, Zaire ebolavirus, GenBank: AIO11753.1), was synthesized by GenScript and cloned into pcDNA3.1(+). Both plasmids were co-transfected into 293 T cells in a 10 cm plate using transfection reagent polyethyleneimine (PEI). Three days post transfection, the medium was harvested and centrifuged at 500 g to remove cell debris, and then the supernatants were stored at −80°C (Platt et al., 2009; Wang et al., 2022). The viral titers were determined by reverse transcriptase assay.

### 2.8 Reverse transcriptase assay

The titers of pseudotyped viruses were determined by Reverse transcriptase assay (RTA) (Wei et al., 2019). 500 μL of pseudotyped virus stock was spun at 14,000 g for 2 h at 4°C to precipitate the virus. The viral pellet was resuspended in a Triton X-100-based suspension buffer and vortexed, followed by three rapid freeze–thaw cycles to lyse the viruses. 50 μL of reaction mix [Oligo-dT Poly-A and ^3^H-dTTP (PerkinElmer)] was added, and the samples were incubated at 37°C for 1 h in a heating block. Then, the samples were pipetted onto DEAE Filter mat circle papers (PerkinElmer), followed by three 10 min washes in 2X SSC buffer, and one 10 s wash in 100% ethanol. The filters were dried at room temperature and analyzed using a scintillation counter to measure the incorporation of ^3^H-dTTP into cDNA. The average CPM values from duplicates were determined.

### 2.9 Virus adsorption

Pseudotyped Ebola virus stocks were mixed with wild-type bacteria or engineered bacteria (~5×10^7^/mL) in 1.5 mL micro-centrifuge tubes. The mixtures of bacteria and viruses were incubated for 1 h at room temperature. Then the tubes were spun for 1 min at a 13,000 g to remove the bacteria and bound pseudotyped virus. The supernatants were collected, and the viral titers determined by RTA.

### 2.10 Virus neutralization

For the neutralization assay, pseudotyped Ebola virus was mixed with wild-type or engineered bacteria in the same manner as the adsorption assay described above. After centrifugation to remove the bacteria and bound pseudovirus, the remaining supernatants were applied to TZM-bl cells which were used as the target cells due to their ability to express luciferase when infected (Platt et al., 2009). The TZM-bl cells were set at a density of 6.0 × 10^3^ per well in a 96-well plate. Each neutralization assay was performed in triplicate with 5,000 RT units of pseudovirus per well used as the starting titer. Two days post-infection, the supernatants were removed, the cells were washed once with PBS, lysed in 1x Passive Lysis Buffer, and frozen at −80°C. The plates were then thawed, and luciferase activity was measured using beetle luciferin substrate (Promega) in a Veritas Luminometer.

### 2.11 Statistical analyses

Statistical analyses were conducted for virus adsorption and virus neutralization data using GraphPad Prism software (version 9.0). The significances were determined by using unpaired two-tailed Student’s *t*-test at *p*-value ≤0.05.

## 3 Results

### 3.1 SVN gene cloning and expression

**Figure 1:**
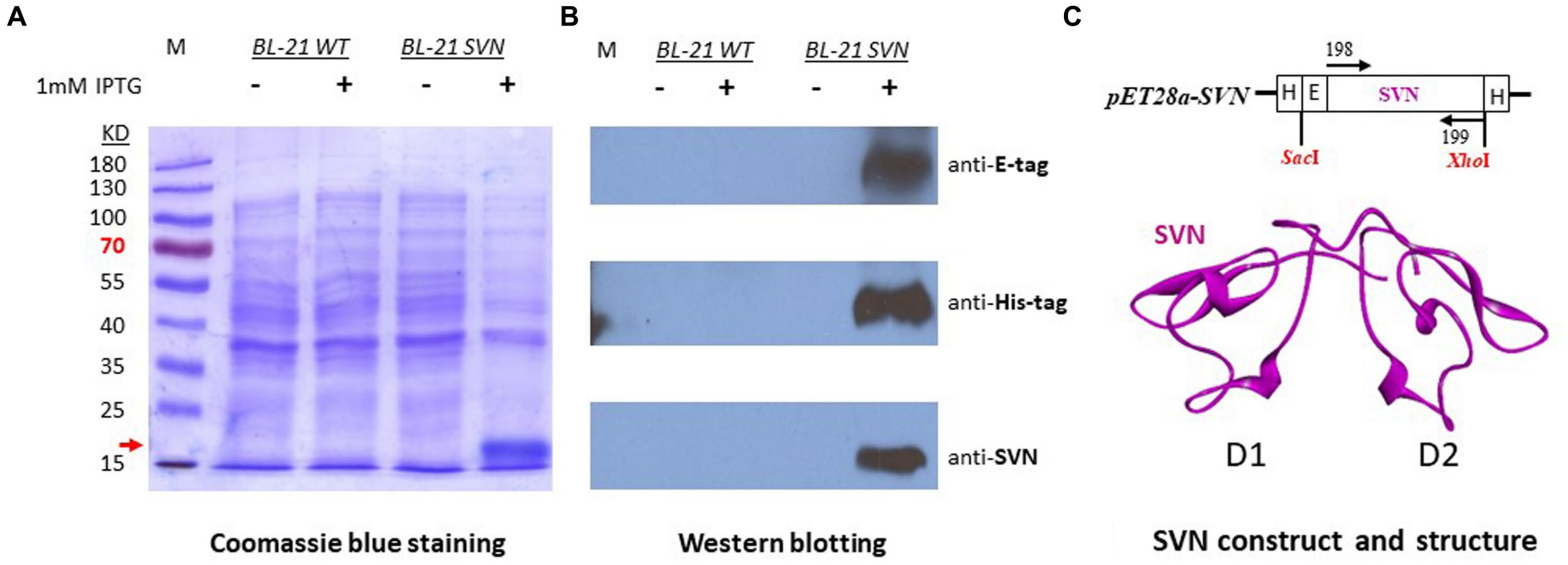
Expression of SVN-E-tag-His-tag in bacterial *Escherichia coli K12 BL21* cells. **(A)** Coomassie blue staining, the induced fusion protein band was marked with the red arrow. **(B)** Western blots showing the specific positive bands. The specific antibody for anti-SVN is a scytovirin polyclonal antibody. **(C)** The SVN construct in pET28a and the SVN 3D structure, showing the two similar domains (D1 and D2) in magenta. H, His-tag, E, E-tag.

The scytovirin gene was initially synthesized by adding an E-tag at the N-terminus and cloned into pET28a with *SacI/XhoI* sites. The SVN plasmid was transformed to *E. coli* BL21 (DE3) cells for protein expression. The SVN fusion protein was observed under induction of 1 mM IPTG by Coomassie blue staining. The induced band of ~16kD fusion protein was noticed clearly (Figure 1A). To further confirm this protein, Western blotting was carried out by using E-tag, His-tag, and SVN specific antibodies. Positive bands were observed from all three different specific antibodies (Figure 1B), suggesting that the ~16kD SVN-E-tag fusion protein is expressed correctly. Figure 1C shows the construct design including the two His-tags encoded by the pET28a vector, as well the SVN ribbon structure with two highly similar domains (D1 and D2) for carbohydrate binding (Moulaei et al., 2007). Thus, the SVN-E-tag expressing construct can be further utilized for following bacterial engineering.

### 3.2 SVN gene engineering for surface display on *Lactobacillus casei*

**Figure 2:**
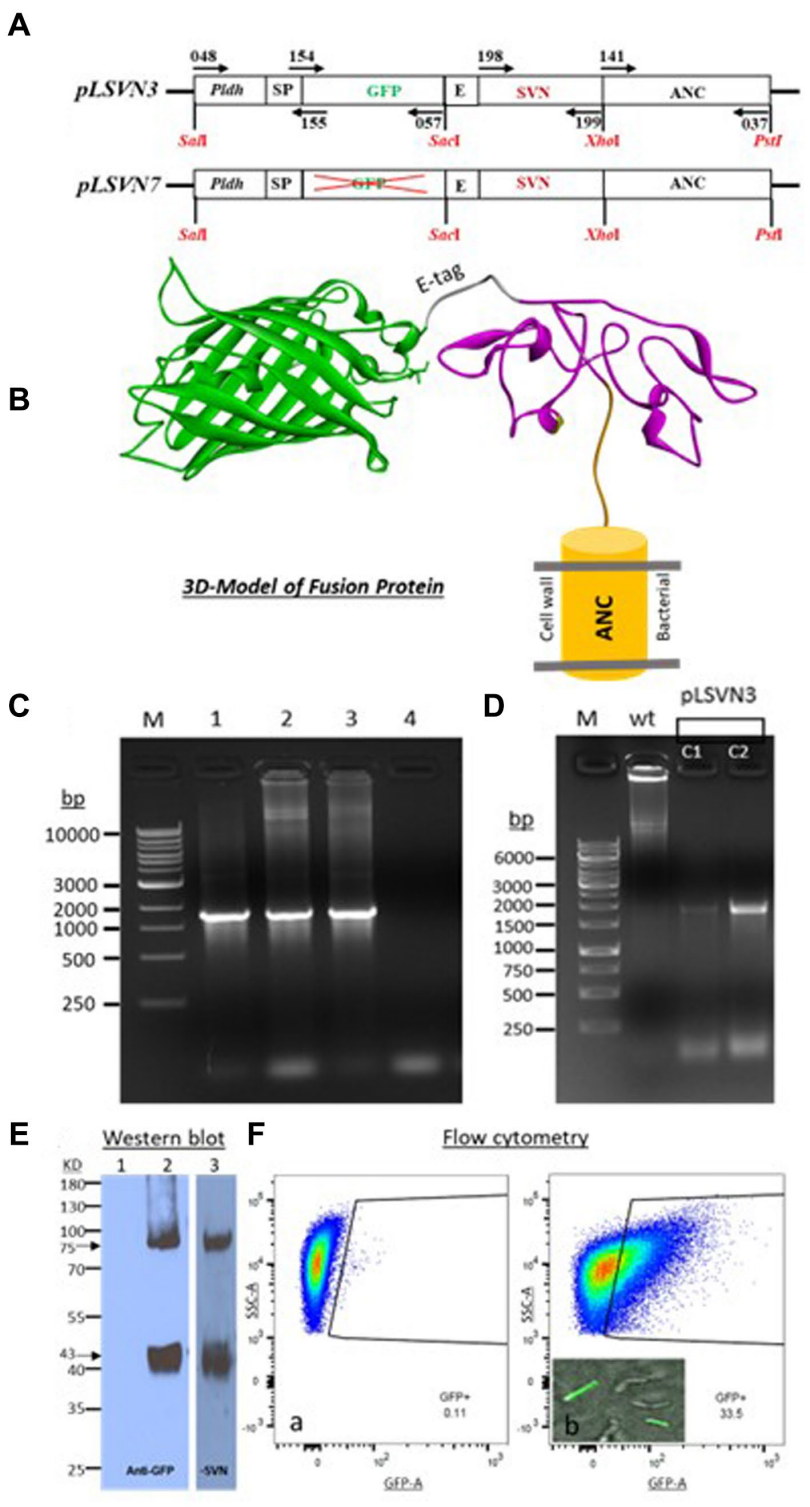
Plasmid constructs and characterizations of SVN-fusion protein expression in *Lactobacillus casei*. **(A)** Maps of scytovirin (SVN) plasmid constructs. Promoter, *Pldh*; SP, signal peptide; GFP, green fluorescent protein; E, E-tag; SVN, scytovirin; ANC, anchor domain. **(B)** Three-dimensional (3D) model of fusion protein of ANC-SVN-E-tag-GFP. **(C)** DNA gels showing the PCR bands with the pair of primers 154/037, indicating the total 1797 bp band from the positive colonies of *E. coli*: 1. pLSVN3 plasmid, 2. pLSVN3 C1 (colony 1), 3. pLSVN3 C2 (colony 2). 4. *E. coli* WT. **(D)** Verification of pLSVN3 plasmid in *L. casei* after electroporation using PCR primers 154/037. wt, wild-type *L. casei*; 1. pLSVN3 C1 (colony 1); 2. pLSVN3 C2 (colony 2). **(E)** Western blot for detecting the SVN-GFP fusion protein. 1. *L. casei* wild-type bacteria only. 2. Engineered *L. casei* (using anti-GFP antibody). 3. Engineered *L. casei* (using anti-SVN HRP conjugated polyclonal antibody, MBS7005164). **(F)** Flow cytometry analysis: (a), Wild-type *L. casei* (negative control), (b). Engineered *L. casei* and the images of SVN-GFP surface displayed bacteria.

**Figure 3:**
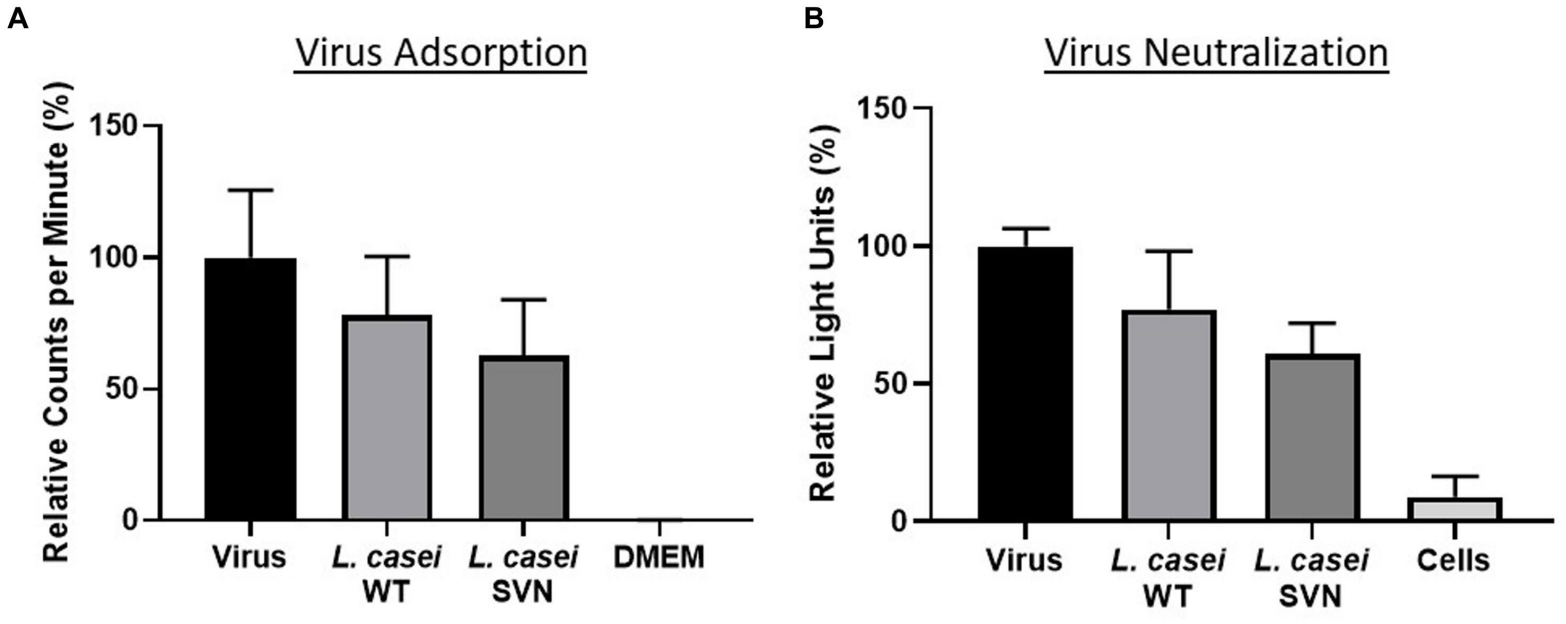
Functional studies of SVN-engineered *L. casei* bacteria. **(A)** Virus adsorption assay. **(B)** Virus neutralization assay. Wild-type (WT) *L. casei* as the negative bacterial control; virus only used as the positive control; DMEM medium or cells as the negative control.

The SVN-E-tag construct was cloned into our previous plasmid (pWZ486) through *SacI/XhoI* sites to produce a new construct designated as pLSVN3 (Figure 2A). This GFP-E-tag-SVN fusion protein with the anchor has been modeled and shown in Figure 2B, which should be flexible for capturing viral particles. The construct was created first in *E. coli DH5α* cells and demonstrated the PCR fragment of ~1800 bp of GFP-E-tag-SVN sequence was correctly amplified using primers 154 and 037 (Figure 2C). To engineer the *L. casei* strain, the SVN construct pLSVN3 was transformed by electroporation into the *L. casei* for protein expression. The transformants were verified by PCR. A ~ 1800 bp PCR band from the amplification with primers 154 and 037 revealed that the pLSVN3 plasmid was transformed into the *L. casei* cells (Figure 2D). A ~ 75kD band corresponding to the fusion protein size (GFP-E-SVN-ANC) was detected by Western blotting using specific GFP and SVN antibodies (Figure 2E), suggesting that the SVN-GFP based fusion protein was produced from the *L. casei* cells. The smaller size band (~43kD) that appeared is the partial fragment of the fusion protein complex without the anchor domain (ANC). Flow cytometry analysis revealed the rate of SVN-GFP positive cells was 33.5% (Figure 2F a and b). To determine whether these protein inhibitors are displayed on the bacterial surface, confocal microscopy was used to visualize these SVN-GFP fused proteins. The pictures from confocal microscopy exhibited the fusion protein in green (GFP) displayed on the surface of bacteria (Figure 2F b). The biological functional studies were conducted for virus binding and neutralization. The engineered bacteria showed a moderate binding activity to the pseudotyped Ebola particles, reduced 37.2% of virus load, but the wild-typed (WT) bacteria also showed weak binding to the pseudotyped viruses because of the unspecific binding, reduced 21.8% (Figure 3A). The virus inhibition assay indicated that SVN-expressing bacteria had a moderate inhibition activity against pseudotyped Ebola virus infection which was 39.1%, but the WT-bacteria also had 23% inhibition (Figure 3B). These moderate functions may be due to the lower positive rate of SVN-expressing cells and the GFP interference of SVN binding in the SVN-GFP-ANC fusion protein complex.

### 3.3 SVN gene engineering for surface display on *Lactococcus lactis*

**Figure 4:**
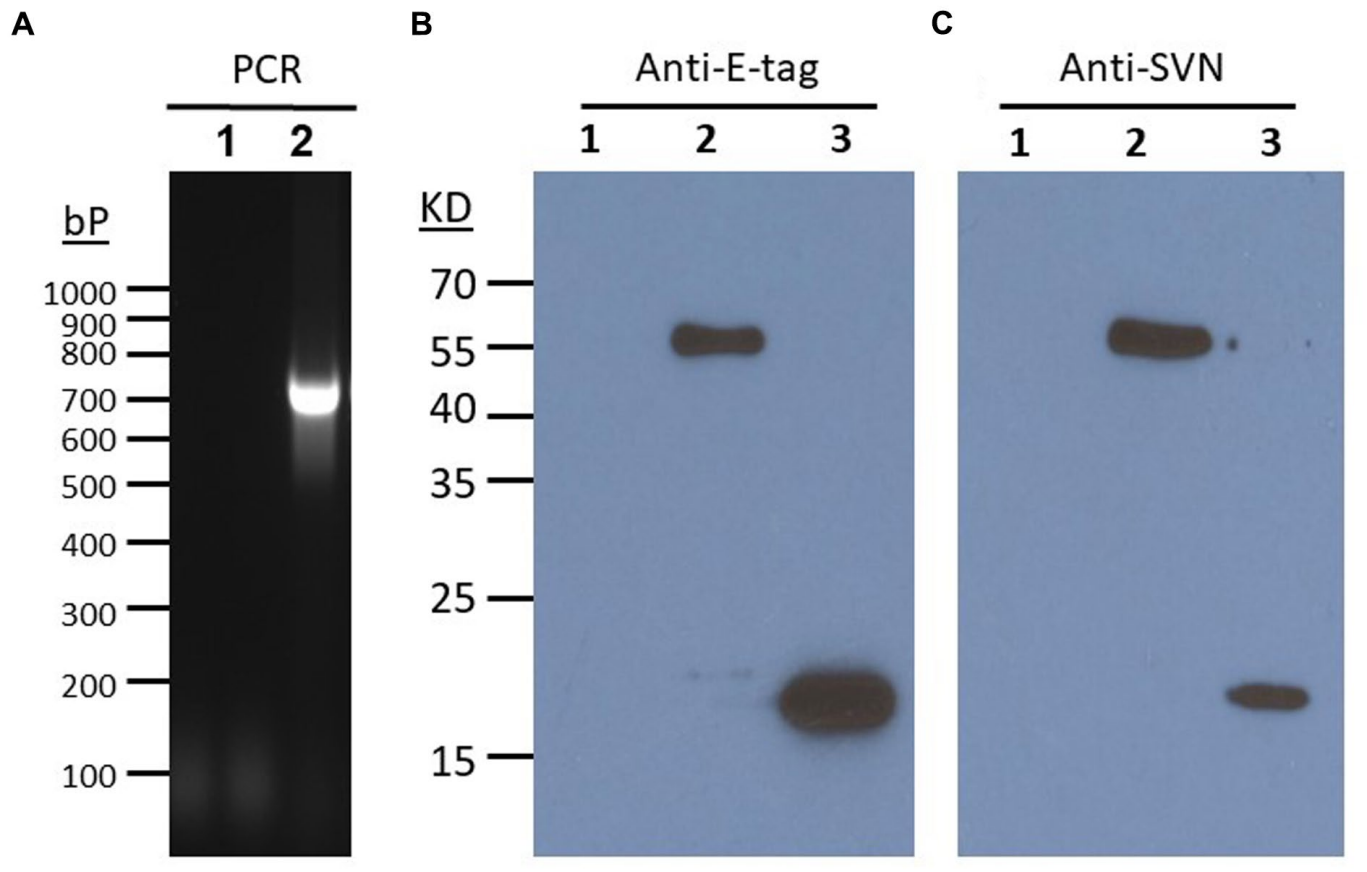
Characterizations of SVN expression in *L. lactis*. **(A)** PCR verification of SVN plasmid in *L. lactis*. PCR verified a positive band of ~700 bp from primers of 048 and SVN reverse primer 199. **(B)** Western blots showing anti-E-tag and Anti-SVN positive bands. 1. *L. lactis* WT, 2, *L. lactis* SVN. 3. *E. coil* SVN (positive control). SVN specific polyclonal antibody A64238-050 (Epigentek).

**Figure 5:**
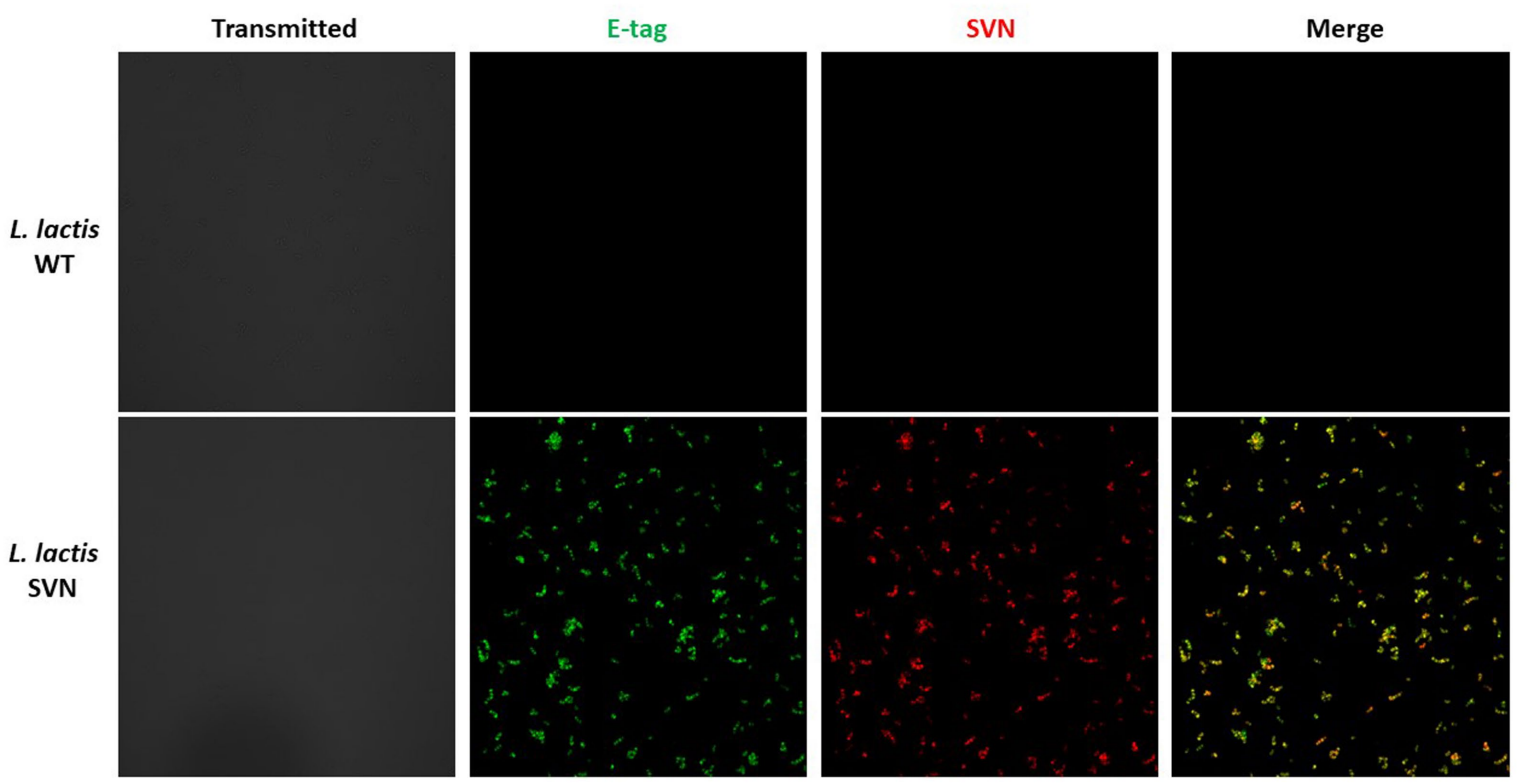
Confocal analysis of SVN-engineered *Lactococcus lactis*. Antibodies used for staining: AF-488 (green) for E-tag; AF-594 (red) for SVN. For more details, please see the Method section.

**Figure 6:**
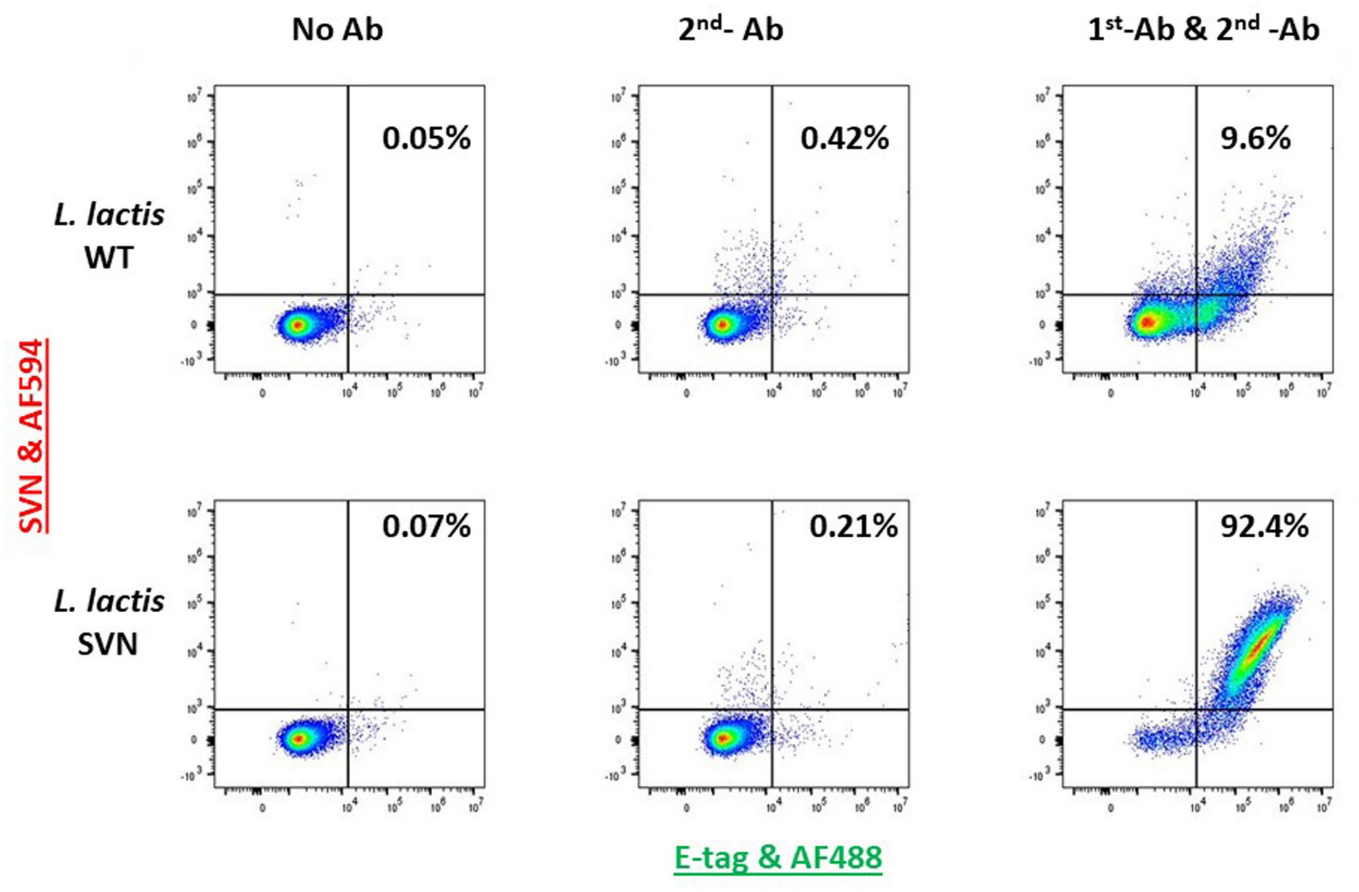
Flow cytometry analysis of SVN-engineered *Lactococcus lactis*. The 1st antibody (AF-488 conjugated, green) used is for staining E-tag; The 2nd antibody used (AF-594, red) is for staining SVN.

*Lactococcus lactis* is another type of LAB which has a round shape and is widely used in the production of buttermilk and cheese. Thus, it is a very safe host strain candidate for anti-Ebola infection. We created the construct to remove the protein marker GFP but keep the E-tag marker from the construct pLSVN3, and this new construct was designated as pLSVN7 (Figure 2A). The construct pLSVN7 was transformed into the *L. lactis* using electroporation method. Positive bacterial colonies in the erythromycin selection plates were picked for further evaluation. The PCR method was first used to confirm the pLSVN7 plasmid was in the bacterium of *L. lactis* using primers 048 and 199. The expected ~700 bp band was identified (Figure 4A). Western blotting further demonstrated that SVN fusion protein (E-tag-SVN-ANC) was shown and in the correct molecular weight of 55kD both in anti-E-tag and anti-SVN specific antibodies. The 16kD SVN-E-tag fusion protein from the *E. coli* BL-21 (DE3) lysate as the positive control was also shown in the Western blots (Figures 4B,C). These Western blotting data demonstrated that the E-tag-SVN-ANC fusion protein was expressed in *L. lactis*. Next, the Confocal microscopy method was used to verify the surface display. Two colors of fluorescence were used for labeling E-tag (green) and SVN (red), respectively. The results demonstrated that the SVN fusion protein is clearly expressed on the surface of the bacterium. The single color, green or red and the merged color of green and red indicated the overlapping presence of fusion protein surface expression (Figure 5). Furthermore, Flow cytometry analysis was also conducted, and the data presented in Figure 6, shows that the positive rate of bacteria has reached 92.4%. The results demonstrated that the SVN-fusion protein is unambiguously expressed on the surface of these bacteria.

**Figure 7:**
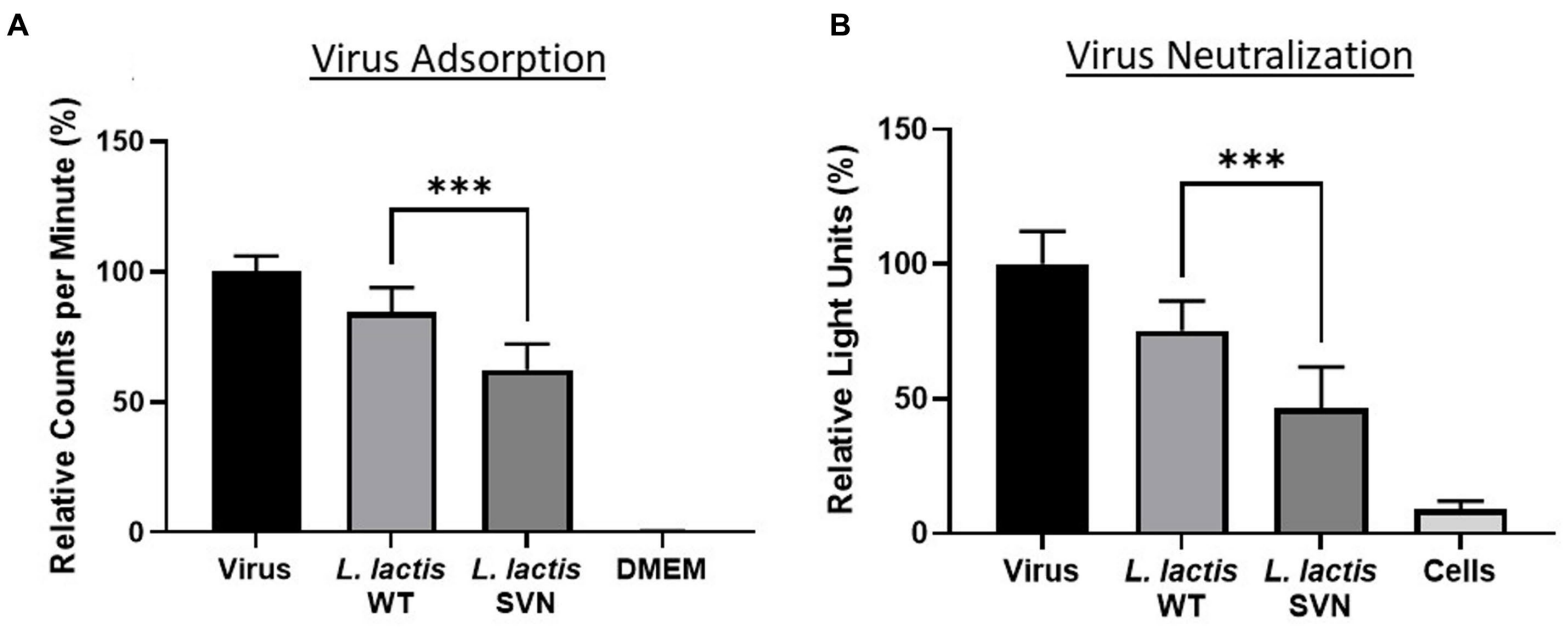
Functional studies of SVN-engineered *L. lactis*. **(A)** Virus adsorption assay. **(B)** Virus neutralization assay. Wild-type (WT) *L. lactis* as the negative bacterial control; virus only used as the positive control; DMEM medium or cells as the negative control. Significances were determined by using unpaired two-tailed Student’s *t*-test at *p*-value ≤0.05.

Functional studies were performed against the pseudotyped Ebola viruses. First, we used the viral particles adsorption method to evaluate the bacterial ability for capturing the viruses. We then tested the engineered strain activity to inhibit Ebola virus infection using TZM-bl cells. The virus adsorption and neutralization data are shown in Figure 7. Compared to the wild-type control bacteria, *L. lactis*, SVN engineered bacteria demonstrated improved functional abilities, absorbing 22.1% more pseudoviral particles and reducing infection by 28.5%. Overall, the SVN expressing *L. lactis* was able to adsorb 37.6% and neutralize 53.5% of the total pseudotyped Ebola virus, reducing infection in half compared to the virus only positive control (Figures 7A,B). In comparison, the wild-type *L. lactis* also demonstrated some ability to bind pseudotyped Ebola virus and reduce infection (15.5% adsorption and 24.7% neutralization) through non-specific binding, but the specific binding of the engineered bacteria to the viruses and are more than twice as effective, suggesting the SVN-expressing *L. lactis* bacteria can offer better protection against Ebola virus infection.

## 4 Discussion

Bacterial therapy is a promising approach for human health and the common use of probiotics demonstrates its great potential (Yadav et al., 2020; Tegegne and Kebede, 2022). Using commensal bacteria for treating viral diseases has received broad attention since the bacterial microbicide has its advantages such as easy-to-use, cost-effective, and long-term efficacy (Ramachandran and Shanmughavel, 2009; Obiero et al., 2012). Especially, natural lectins as inhibitors can reduce unnecessary immune responses and enhance specific inhibiting activities for therapeutic applications (Akkouh et al., 2015; Mitchell et al., 2017; El-Maradny et al., 2021; Fernandez Romero et al., 2021; Mazur-Marzec et al., 2021; Naik and Kumar, 2022). For developing this method to use bacteria for antiviral diseases, bacterial engineering is a critical step. In this report, we engineered two types of Lactic acid bacteria (LAB) for surface display of the Ebola inhibitor scytovirin (SVN). Both bacterial strains successfully displayed scytovirin on the surface, suggesting the applicability of our constructs for surface expression of SVN fusion protein in lactic acid bacteria. The fluorescence of the SVN lectin fusion molecules when stained with GFP or SVN antibodies perfectly overlapped on the surface of the bacterium indicating a high display of inhibitors. The positive rate of engineered bacteria is higher in *L. lactis* (92.4%) than in *L. casei* (33.5%), suggesting that lower positive rate in *L. casei* is due to the higher genetic instability because certain bacteria may lose the SVN plasmid or SVN gene during the replication, especially when the antibiotic pressure is reduced. Another possibility is the genetic recombination between the SVN plasmid and host genome by which the plasmid composition could be changed or damaged. We found that LAB were capable of quickly developing resistance to erythromycin (5 μg/mL) selective pressure, especially when grown in stationary, liquid culturing conditions, suggesting that the bacteria lose the plasmid (data not shown). Thus, increased erythromycin concentration is usually needed to get more genetically stable strains. In general, using a genomic integration method for engineering would be better than plasmid transformation for making genetically stable strains, which is essential for clinical or therapeutic use. In this research, *L. lactis* was found to be highly stable and would be assessed for *in vivo* (mice) colonization, and for protective efficacy against challenge with infectious Ebola viruses conducted in the BSL-4 containment.

In the construction of fusion protein plasmids, the GFP protein marker seems unnecessary. Small protein tags such as E-tag appear to be adequate for detection and evaluation during the studies. Removing GFP reduced the size of the fusion protein by ~27kD, nearly 3x the size of SVN, maximizing the exposure of SVN inhibitor and preventing possible steric hindrance from GFP. Eliminating GFP from the fusion protein also presents another advantage for *in vivo* applications by avoiding the potential for immunogenicity and cytotoxicity caused by the GFP protein (Ansari et al., 2016).

In this report, our data from the *in vitro* study with pseudotyped Ebola virus serves as a proof of concept of using engineered commensal bacteria for blocking Ebola infection. Commensal bacteria have previously been demonstrated to inhibit HIV-1 infection (Nahui Palomino et al., 2017), and it is likely they provide a similar baseline level of protection at mucosal surfaces against other viral infections. This study indicates that this baseline level of protection is around 20–25% for WT *L. lactis*. Our data shows that lectin displaying bacteria can bind and neutralize significantly more viruses compared to the wild-type commensal bacteria, suggesting that engineered bacteria could be used prophylactically to improve upon the health benefit already provided by commensal bacteria by decreasing the likelihood of contracting a viral disease. This is contingent upon the engineered bacteria successfully colonizing various mucosal surfaces in the body and stably producing recombinant protein. To investigate this, we will test different routes of administration in mice (oral, nasal, rectal, etc.) to determine the stability of the *L. lactis* strain under different physiological conditions, as well as determining the efficacy of the bacteria to prevent *in vivo* infection prior to advancing to clinical trials. In conclusion, the commensal bacterial based anti-Ebola approach is promising and will be beneficial for combating this deadly viral disease.

## Data availability statement

The datasets presented in this study can be found in online repositories. The names of the repository/repositories and accession number(s) can be found in the article/supplementary material.

## Ethics statement

Ethical approval was not required for the studies on humans in accordance with the local legislation and institutional requirements because only commercially available established cell lines were used. Ethical approval was not required for the studies on animals in accordance with the local legislation and institutional requirements because only commercially available established cell lines were used.

## Author contributions

JW: Data curation, Methodology, Writing – review & editing, Formal analysis, Investigation, Validation, Visualization. NN: Data curation, Formal analysis, Investigation, Methodology, Validation, Writing – review & editing, Visualization. WW: Data curation, Formal Analysis, Investigation, Methodology, Writing – review & editing. LW: Formal Analysis, Investigation, Methodology, Writing – review & editing. HH: Investigation, Writing – review & editing. S-HX: Data curation, Investigation, Writing – review & editing, Conceptualization, Formal Analysis, Funding acquisition, Methodology, Project administration, Resources, Supervision, Writing – original draft.
